# Study of the digital model of self-regulation system of the heart

**DOI:** 10.1186/1878-5085-5-S1-A89

**Published:** 2014-02-11

**Authors:** Anatoliy O Gorbach, Rostyslav V Bubnov

**Affiliations:** 1 Ukrainian Academy of Informatics, Ukraine, Kyiv, Ukraine; 2 Clinical Hospital “Pheophania” of State Affairs Department, Kyiv, Ukraine

## Introduction

Promoting PPPM calls the new demands for mathematical models in medicine that have to reflect the similarity of structure and function of the original system, should be always simpler and have little distorts the original. Simplification is necessary because of the greater complexity of living systems, and the distortion depends on the method of simulation. With development of cardiology technologies relevant models of the cardiovascular system, taking into account the regulatory processes should be implemented.

## The aim

of the paper was to study the mathematical model of the system of self-regulation of the heart.

## Materials and methods

The mathematical equation (1) allows us to consider the work of such a system is dependent on the values of the coefficients.(1)

where: *V_1_* - heart ventricle filling, *V_2_* - stroke volume, *P_2_* - the average pressure in aorta, *Vres* - residual blood volume, *V_n_* - the cavity volume which “heterometric dependence” of the heart function starts from, *K* - pump ventricular coefficient, *K_7_* - depending on the inertia heterometric ratio, *K_8_* - the coefficient of inertia of “heterometric dependence”

We estimated parameters and variables obtained from healthy volunteers.

## Results

Analysis of the results show that the reaction model for “jumping” changes of flow (3 ÷ 125) and *K_7_* = *K_8_* = 0 is very short-lived, if 0.4 ≤ *K* ≤ 0.9 and can be up to 50 cycles (25 seconds) for *K* close to 0.1. The form has a smooth transient nature of overshoot is observed. The results were similar for different signs of stress (+122, -122) and the magnitude of the perturbation.

*K_7_* factor in normal physiological conditions, may lead to higher performance, due to the deregulation and subsequent asymptotic approximation to the steady value. The transition process in this case almost always ends in 2 ÷ 6 cycles. If *K_7_* is less than 0.5, the control error, is less than 25% even at the maximum of unfavorable perturbation (kind of trigger effect - acceleration curve).

**Fig. 1:**
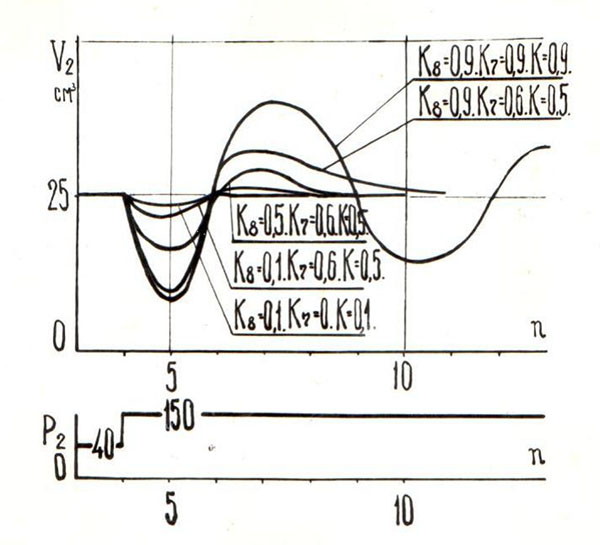
the reaction of the model to a step of pressure change

For a perturbation of the system of self-regulation of the heart are associated with changes in blood pressure, the coefficient *K* does not bind significantly to the transition process. The parameter that determines the heterometric depending inertia (*K_8_*), affects the output of the system time to a steady value (fig. 1). If *K_8_*> 0, 5, then the observed oscillatory process is not consistent as physiological representations [3]. The coefficient *K_7_* has very little effect on the system when the pressure in the aorta.

## Conclusions

The inertia related to both heterometric and gemeometrical properties play a significant role in the dynamics of the heart. Using a mathematical model of the cardiovascular system with implemented regulation processes monitoring, and real-time operating computer systems can support personalized therapy. Similarly, qualitative analysis is required to select the appropriate therapy.

## Outlook and expert recommendations

We recommend to implement self-regulation models of the heart that can be useful when choosing personalized therapeutic approach, considering compensatory, homeostatic and protective reaction of the organism, can predict concomitant complications.
